# Silver nanoparticle incorporation effect on mechanical and thermal properties of denture base acrylic resins

**DOI:** 10.1590/1678-775720160185

**Published:** 2016

**Authors:** Ayşegül KÖROĞLU, Onur ŞAHİN, Işın KÜRKÇÜOĞLU, Doğu Ömür DEDE, Tonguç ÖZDEMİR, Baki HAZER

**Affiliations:** 1 - Bülent Ecevit University, Faculty of Dentistry, Department of Prosthodontics, Zonguldak, Turkey.; 2 - Süleyman Demirel University, Faculty of Dentistry, Department of Prosthodontics, Isparta, Turkey.; 3 - Ordu University, Faculty of Dentistry, Department of Prosthodontics, Ordu, Turkey.; 4 - Mersin University, Faculty of Engineering, Department of Chemical Engineering, Mersin, Turkey.; 5 - Bülent Ecevit University, Faculty of Arts and Sciences, Department of Chemistry, Zonguldak, Turkey.

**Keywords:** Nanoparticles, Acrylic resin, Flexural strength, Impact strength, Differential scanning calorimetry

## Abstract

**Objective:**

The aim of the present study was to evaluate the mechanical and thermal characteristics of two denture base acrylic resins containing silver nanoparticles (AgNPs).

**Material and Methods:**

Two different acrylic denture base resins (heat-polymerized and microwave polymerized) containing 0.3, 0.8 and 1.6 wt% AgNPs were evaluated for flexural strength, elastic modulus and impact strength. The glass transition temperature (Tg) and relative heat capacity (Cp) of the samples were determined from the Differential Scanning Calorimetry (DSC) results. For statistical analysis, two-way ANOVA and Tukey-HSD tests were performed.

**Results:**

Addition of 0.8% and 1.6% AgNPs in microwave-polymerized resin significantly decreased the transverse strength and elastic modulus. In terms of impact strength, the addition of AgNPs has no effect on both resin groups. Glass transition temperature (Tg) was decreased with the addition of AgNPs for both denture base resins.

**Conclusions:**

The incorporation of AgNPs, generally used for antimicrobial efficiency, affected the transverse strength of the denture base acrylic resins depending on the concentration of nanoparticles. Tg was decreased with the addition of AgNPs for both denture base resins.

## INTRODUCTION

Poly(methyl methacrylate) (PMMA) is widely used in the preparation of partial and total denture bases. The proliferation of certain pathogens such as *Candida albicans* and *Streptococcus mutans* is induced by the surface roughness of acrylic resins and local or systemic factors^1^ ^,^ ^6^ ^,^ ^25^. The improvement of oral hygiene is generally achieved by the use of antimicrobial mouthwashes and appropriate tooth-brushing methods along with the use of denture cleansing tablets and prophylactic systemic antibiotics. However, all these methods have limited success in reducing the effectiveness of these pathogens^7^ ^,^ ^19^. For these reasons, research on broad-spectrum antimicrobial acrylic resin materials has attracted much interest in recent times^13^.

Silver (Ag) salts have been used for thousands of years, because of their antimicrobial efficiency against Gram-positive and Gram-negative bacteria, protozoa and fungi, as well as viruses^18^. Nowadays, elemental Ag and associated compounds are used to reduce the risk of infection in the treatment of burns, prevent bacterial colonization on medical devices, in surgical textile fabrics, for water purification, bone cements, and dental materials^11^ ^-^ ^14^ ^,^ ^18^ ^,^ ^24^.

In dental applications, different forms of Ag such as Ag ions (Ag^+^), Ag nanoparticles (AgNPs), and Ag-polymeric complexes have been used to improve antibacterial efficiency^7^ ^,^ ^16^. The instability of Ag^+^, however, restricts its practical implementation. The problem can be resolved by protecting the Ag^+^ with a polymeric matrix sheath. The major advantage of using AgNPs arises from their large ratio of surface area to volume. AgNPs exhibit more effective ion release and enhanced antimicrobial activity^5^. AgNPs are preferred for this reason, alongside additional functional assets such as their ductility, electrical and thermal conductivity^7^ ^,^ ^17^ ^,^ ^22^ ^,^ ^25^.

Although the antimicrobial characteristic of AgNPs in acrylic resins has been previously illustrated^1^ ^,^ ^13^ ^,^ ^19^ ^,^ ^23^ ^,^ ^27^, there are few studies reporting the influence of NPs on the mechanical properties of denture base resins^10^ ^,^ ^13^ ^,^ ^25^. Although the addition of AgNPs has antimicrobial advantages on acrylic resins, its effect on the mechanical properties of the resin should be examined^25^. Therefore, the aim of this *in vitro* study was to evaluate the effect of AgNPs on the flexural strength, elastic modulus, impact strength, and differential scanning calorimetry (DSC) properties of two distinct dental acrylic resins.

## MATERIAL AND METHODS

Two acrylic resins used in this study were; (1) conventional heat-polymerized PMMA resin (Meliodent, Bayer Dental, Berkshire, UK); and (2) microwave-polymerized PMMA resin (Acron MC, GC Dental, Tokyo, Japan).

### Synthesis and characterization of AgNPs

The AgNPs were prepared based on the Turkevich method^26^. Silver nitrate (AgNO_3_) was dissolved in water (208 mg AgNO_3_/100 mL H_2_O) and the solution was brought to a boil (at 100°C). After 2 min of boiling, an aqueous solution of sodium citrate (Na_3_C_6_H_5_O_7_) (49 mg sodium citrate/1.25 g H_2_O) was added. The formation of AgNPs was perceived from the emergence of a yellow colour in the previously colourless solution. The solution was boiled further for another 6 min and then allowed to cool. Ultraviolet (UV) visible absorption spectroscopy (T80+, PG Instruments, Leicester, UK) and Transmission electron microscopy (TEM) (Technai G^2^ Spirit BioTWIN, FEI, OR, USA) were used to characterize the formation of AgNPs. Also, a Zeta Sizer Instrument (Malvern Instruments Ltd., Malvern, UK) was used to determine the particle size. Distilled water was used as a dispersion media during the particle size-determination process.

### Specimen preparation

In total, 56 specimens (n=7) were prepared for flexural and impact strength tests, with dimensions of 65×10×2.5 mm and 50×6×4 mm, according to the ADA Specification No.12 and ISO/DIS 1567:1998 standards, respectively. The powder/liquid ratios for heat-polymerized and microwave-polymerized resin were 35 g/14 mL and 100 g/43 mL, respectively. For the study groups to determine the effect of AgNPs, the suspension of AgNPs was mixed with each resin monomer in concentrations of 0.3, 0.8, and 1.6 wt% and sonicated for 15 min. Afterwards, the liquid component was mixed with the powder part, in accordance with the manufacturer’s instructions. The heat-polymerized specimens were cured in a water bath at 100°C for 30 min while the microwave-polymerized specimens were irradiated for 3 min at 600 W. Before deflasking, all the specimens were bench-cooled. Test specimens were wet ground with silicone carbide grinding papers of 200, 400, and 600-grit sizes using an automatic polishing machine (Grin PO 2V grinder-polisher, Metkon A.Ş., Bursa, Turkey). Before testing for full saturation, the flexural test specimens were kept in distilled water at 37°C for 50±2 h, and impact test specimens were stored at 37°C for 2 weeks^8^ ^,^ ^15^.

### Flexural strength testing

The flexural strength test was performed by using a Lloyd universal testing machine (Lloyd Instruments, LRX, Fareham, UK) with a crosshead speed of 5 mm/min. Flexural strength (FS) was determined using the following formula;

FS=3Fl/2bh^2^

where F is the maximum load applied (N), l is the distance between supports (span length=50 mm), b is the width of the specimen (10 mm) and h is the thickness of the specimen (2.5 mm).

Elastic modulus (E) was calculated from the formula;

EM=Fl^3^/4bh^3^d

where d (mm) is the deflection.

### Impact strength testing

Impact strength test was performed using a Charpy-type impact tester (Coesfeld, Pendulum Impact Tester, Dortmund, Germany). Impact strength (IS) was calculated using the following formula;

IS=E/wt

where E is the energy required to break the specimen (J), w is the width (6 mm) and t is the thickness of the specimen (4 mm).

### Thermal analysis

Differential Scanning Calorimetry (DSC) was performed with a Perkin Elmer Pyris 1 instrument and the analyses were carried out within a temperature range of 30°C to 200°C at a heating/cooling rate of 20°C/min; Nitrogen was used as an inert atmosphere. 6 mg of sample was used for the DSC analysis and they were placed in an aluminium pan. The glass transition temperature (Tg) was taken as the peak temperature of the glass transition region. Tg and relative heat capacity (Cp) of the samples (control samples and 1.6 wt% AgNPs incorporated into microwave and heat polymerized samples) were determined from the DSC tests.

### Statistical analysis

In each group, the mean and standard deviation values were calculated. For statistical analysis, two-way ANOVA and Tukey-HSD tests were performed. Statistical significance was set at *p*<0.05.

## RESULTS

### Synthesis and characterization of AgNPs

**Figure 1:**
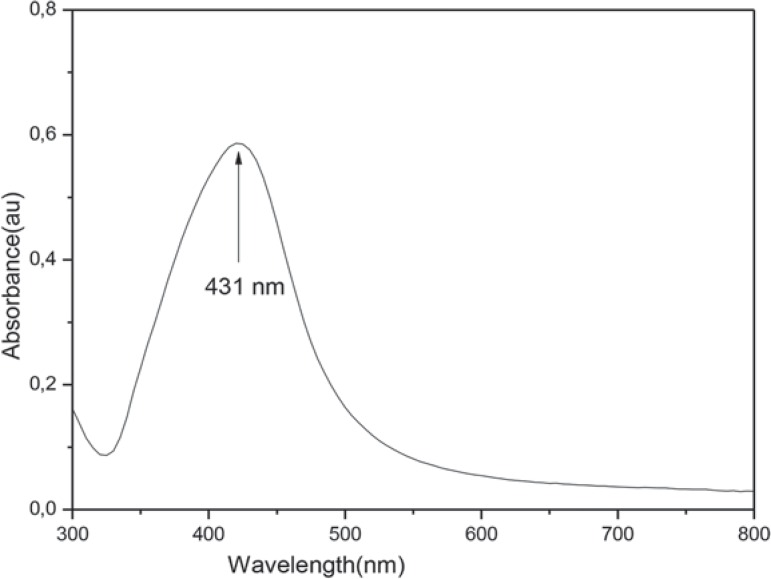
UV absorption spectra of AgNP solution

**Figure 2:**
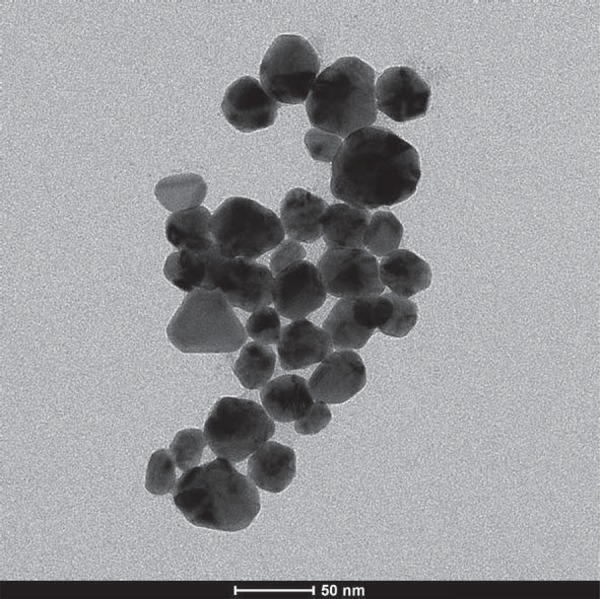
Particle size distribution for the AgNPs

**Figure 3:**
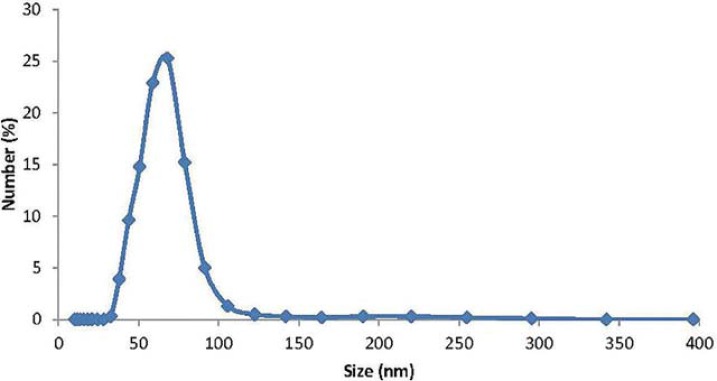
Particle size distribution for the AgNPs

According to the UV visible absorption spectroscopy AgNPs have a UV absorption band with a peak centered around 431 nm (Figure 1). Shape and size distribution of the synthesized AgNPs were characterized by TEM study. The TEM image shown in Figure 2 was obtained by high contrast TEM [FEI Technai G^2^ Spirit Bio (TWIN)]. The TEM image showed that the nano particles have rather similar and mainly spherical-like shapes. The results of the nanoparticle size distribution according to the Zetasizer Instrument showed that the mean nanoparticle size was about 68 nm and was illustrated in Figure 3.

### Flexural strength test results

For each test group, the calculated mean and standard deviation values of flexural strength in Table 1 and the elastic modulus are given in Table 2. Among the eight test groups, the highest flexural strength and elastic modulus values were found in the microwave-polymerized resin group with 0.3 wt% of added AgNPs, while the lowest values were observed for the 1.6 wt% AgNPs-added conventional heat-polymerized resin group. For the conventional heat-polymerized resin group, the addition of AgNPs had no effects on flexural strength and elastic modulus. However, the addition of 0.8 and 1.6 wt% AgNPs in the microwave-polymerized resin significantly decreased the flexural strength and elastic modulus (p<0.05).

**Table 1:** Means and standard deviations of transverse strength for the groups tested (in MPa)

Denture Base Material	Control	0.3 % AgNPs	0.8 % AgNPs	1.6 % AgNPs
Meliodent	104.30 (5.82)^a^	102.71 (3.45)^a^	99.37 (7.94)^a^	97.34 (9.21)^a^
Acron	192.43 (3.05)^c^	197.60 (6.07)^c^	155.01 (5.81)^b^	146.56 (5.29)^b^

Results of Tukey *post-hoc* comparisons were shown as superscripts and values having the same letters do not differ significantly (p>.05)

**Table 2:** Means and standard deviations of elastic modulus for the groups tested (in GPa)

Denture Base Material	Control	0.3 % AgNPs	0.8 % AgNPs	1.6 % AgNPs
Meliodent	1.90 (0.21)^a^	1.87 (0.25)^a^	1.92 (0.24)^a^	1.81 (0.17)^a^
Acron	3.96 (0.58)^c^	4.04 (0.22)^c^	3.02 (0.47)^b^	2.79 (0.67)^b^

Results of Tukey *post-hoc* comparisons were shown as superscripts and values having the same letters do not differ significantly (p>.05)

### Impact strength test results

For each test group, the calculated mean and standard deviation values of impact strength are given in Table 3. The highest impact strength was observed for the conventional heat-polymerized resin without AgNPs and the lowest was for the microwave-resin group with 0.8 wt% AgNPs content. The addition of AgNPs had no effect on the impact strength of both resin groups (p>0.05).

**Table 3:** Means and standard deviations of impact strength for the groups tested (in kJ/m2)*

Denture Base Material	Control	0.3 % AgNPs	0.8 % AgNPs	1.6 % AgNPs
Meliodent	12.32 (0.81)^b^	10.78 (0.72)^ab^	11.64 (1.12)^ab^	11.14 (1.39)^ab^
Acron	10.93 (0.97)^ab^	10.80 (0.97)^ab^	10.35 (0.45)^a^	10.37 (1.23)^a^

Results of Tukey *post-hoc* comparisons were shown as superscripts and values having the same letters do not differ significantly

### Thermal analysis results

**Figure 4:**
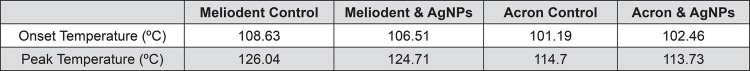
DSC test results

**Figure 5:**
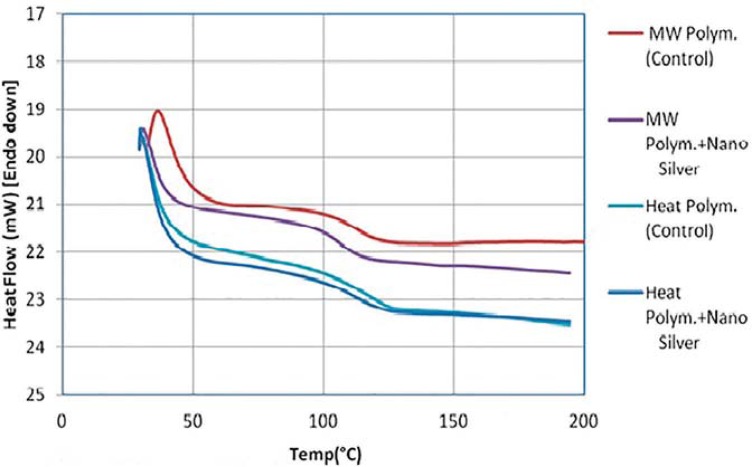
DSC test results - Heat flow

**Figure 6:**
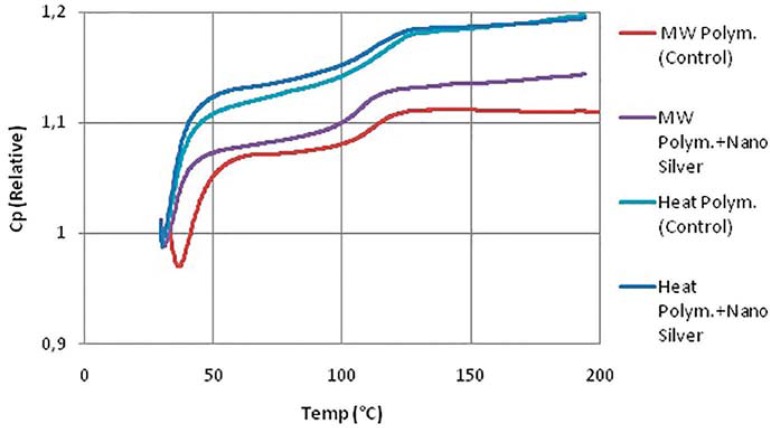
DSC test results - Change of heat capacity (Relative Cp)

The results of the DSC analysis, namely DSC thermograms, onset temperatures of the glass transition and glass transition temperatures (Tg) are given in Figure 4 and Figure 5. It was observed that Tg was higher for the heat-polymerized denture base resin. Tg was found to decrease with the addition of AgNPs for both types of denture base resins. The relative change in heat capacity was calculated from the heat flow data and is illustrated in Figure 6. This was probably due to a decrease in inter and intra-molecular forces within the polymer matrix upon incorporation of the nanoparticles therein.

## DISCUSSION

There are many articles in the literature regarding AgNPs incorporation in denture base acrylic resins^1^ ^,^ ^9^ ^,^ ^10^ ^,^ ^13^ ^,^ ^19^ ^,^ ^25^. In this study, the AgNPs were synthesized by the Turkevich method^26^, through the reduction of silver nitrate with sodium citrate. The nanoparticles are insoluble and their size is typically less than 100 nm^28^. In the present study, the mean particle size determined via Zeta Sizer was about 68 nm. The results demonstrate that nanostructures were achieved and the particle size distribution has a Maxwell-Boltzmann like distribution. Panacek, et al.^21^ (2006) and Baker, et al.^2^ (2005) reported that the smaller particles are more effective against bacteria due to the large surface area. Also, the shape of the nanoparticle affects the antimicrobial efficiency. It was reported that triangular AgNPs expressed greater biocidal activity against *E. coli* than rod- or spherically-shaped nanoparticles^20^. In the current study, as seen in the TEM image (Figure 2), AgNPs are in spherical-like shapes.

For the preparation of nanocomposites, three approaches have been developed: (a) mixing nanoparticles with the polymer, (b) generating nanoparticles during polymerization, (c) adding nanoparticles to the monomer^3^. In the current study, to reduce the agglomeration and to readily achieve polymer/silver nanocomposites, the prepared aqueous solution of AgNPs was dispersed in acryl liquid in the desired ratio and then mixed with the powder part of the acrylic material.

The nanoparticles added to the polymer materials were appropriately quantified such that the quantities used do not cause adverse effects^18^. Yen HJ, Hsu SH and Tsai CL^29^ (2009) reported that depending on the particle size and concentration, AgNPs showed various degrees of adverse effects on macrophages. In a study that investigated the effect of nanosilver on the mechanical and thermal properties of the acrylic base of complete dentures, 5 wt% of nanosilver was added, in order to minimize probable unfavourable changes in the mechanical and chemical properties of the acrylic base of the denture^10^. In another study examining the effect of AgNPs on the mechanical properties of acrylic resins, 0.2 and 0.05 % of AgNPs were used. This study revealed that the effect of AgNPs on the flexural strength of PMMA depended on several factors such as the type of acrylic resin and the concentration of nanoparticles^25^. In the current study, 0.3, 0.8, and 1.6 wt% of AgNPs were used. By utilizing low concentrations of nanoparticles, material costs and the amount of monomer used can be reduced, thus rendering our process cost effective. As a result, the mechanical properties and aesthetic appearance of the cured polymer pose less risk^9^.

In the present study, the interaction between acrylic resin types and the addition of AgNPs at different ratios was not significant for flexural strength, elastic modulus and impact strength tests (p>0.05). Results of this study indicate that the addition of 0.8 and 1.6 wt% AgNPs alters the flexural strength and elastic modulus of microwave-cured PMMA. This may have resulted from the aforementioned AgNPs ratios acting as impurities within the resin, which led to a decrease in the mechanical strength of the polymer. Also, upon adding nanoparticles, the unreacted monomer quantity may be increased depending on the decrease of the monomer reaction and it acted like a plasticizer^9^. For impact strength values, the presence of AgNPs had no effect on the test groups. The study of Sodagar, et al.^25^ (2012) demonstrated that the addition of 0.05% AgNPs caused a decrease in the flexural strength of one brand of self-curing resin but led to an increase in the other brand’s strength. It is hence reported that the type of acrylic resin and the amount of NPs incorporated therein are the important factors which affect the flexural strength of PMMA. In contrast to our study, the study by Kassaee, et al.^13^ (2008) indicated that adding 0.5% AgNPs into the self-curing acrylic resin system increases the flexural strength and antibacterial effect of the material. The differences in acrylic resin type, the amount of NPs, and polar interactions which formed between C=O groups of the PMMA chains and AgNPs, may be responsible for this situation^25^. Chladek, et al.^4^ (2013) reported that the mechanical and physical properties of the final polymer are adversely affected with increasing AgNPs concentration. In a study that evaluated the effect of adding AgNPs to PMMA at two different weight percentages (0.2 and 2 wt%), the following was observed: the effect of AgNPs depended on its ratios, and an increment in AgNPs increased compressive strength but led to a decrease in the tensile strength of the resins. As a result, the final material became more brittle than the pure resin itself^9^. According to the results of the present study, in low concentrations AgNPs, clinically, have no negative effects on the mechanical properties of acrylic resins.

In the present study, the incorporation of AgNPs resulted in a change in thermal properties of the resin. Although the glass transition temperature decreased, the relative decline was not significant enough to sacrifice the thermal stability of the denture base resin. The observed decrease in the glass transition temperature is probably due to an increased discontinuity within the polymer phase and a decrease in the inter- and intra-molecular forces within the polymer matrix upon the incorporation of AgNPs therein. The heat capacity increased within the glass transition region (Figure 6), due to an increment in free volume within the polymeric matrix and second order phase transition in the glass transition region. Increase of the free volume would decrease the heat to be transferred via the conduction mode due to the increase of the intermolecular distances of the polymeric chains.

This *in vitro* study had some limitations. Different nanoparticle concentrations, their respective microbiological aspects and their effects on colour changes should be taken into consideration in future investigations.

## CONCLUSIONS

Within the limitations of this *in vitro* study, the following conclusions were drawn:

Incorporation of 0.8 and 1.6 wt% AgNPs decreased the flexural strength and elastic modulus of microwave-polymerized acrylic resin but had no effect on the other test groups;

For the two resin groups and test specimens, the addition of AgNPs had no effects in terms of impact strength;

The glass transition temperature (Tg) decreased with the addition of AgNPs for both of the denture base resins.
